# Gender budgeting in the post-pandemic period: an analysis in the context of health and safety of women in India

**DOI:** 10.3389/fpubh.2025.1654318

**Published:** 2025-10-07

**Authors:** Kanksha Barman, Indrani Gupta

**Affiliations:** Health Policy Research Unit, Institute of Economic Growth, University Enclave, New Delhi, India

**Keywords:** gender budgeting, health, safety, employment, gender, women’s health

## Abstract

Indian women suffered disproportionately due to the COVID-19 pandemic, with the loss of livelihoods, a steep increase in care work due to lockdowns, and the presence of a deeply patriarchal society that expects women to shoulder the brunt of unpaid domestic labor, as well as increasing incidents of violence. This study analyzes whether the post-COVID gender budgets in India adequately responded to the needs of women highlighted by the pandemic. We examine the six most recent gender budgets of the Government of India to analyze whether appropriate changes were introduced, keeping in mind the impact of COVID-19 on women. We find minimal changes in allocations, with only a slight increase in total allocations over the last two budgets, despite alterations within budget allocations. We find that the bulk of the allocations in the gender budget are limited to a few key ministries, with the main focus of the gender budget having been on rural development, housing, education, livelihoods, health, and nutrition. We also analyzed health, employment, and empowerment indicators for women to examine whether there have been improvements in these areas, and found that while some of the basic health indicators have improved, labor force participation rates and political empowerment of women have declined. We conclude that to actually make a difference to the status of women, one has to go beyond mere participation statistics and look at the needs, concerns, and challenges faced by women. Policymakers need to work with communities and grassroots organizations that work closely with and represent women. A top-down approach with routine budget allocations under the gender budget umbrella might, in fact, do a disservice to the cause by bringing in complacency, which India can ill afford.

## 1 Introduction

There is now a large body of evidence that indicates that the COVID-19 pandemic adversely impacted women socially and economically, though the infection itself may have been gender-neutral. Thus, while men were more prone to the infection and subsequent illness and deaths, women—either directly or indirectly—have borne a heavy brunt of the impact. Studies from across the world have shown that women, and especially mothers, faced a disproportionate burden of care work during the pandemic (1, 2). There are various ways in which the pandemic has affected women’s security globally, with declining accountability for women’s social and private sphere rights, increased discrimination in mobility, informal labor, and healthcare, exacerbated vulnerability of migrants and minorities, and significant amendments to reproductive rights (3). Similar gendered effects have been observed in previous pandemics like the 1918 influenza pandemic and the global Ebola epidemic of 2014–2016 (4). Most reviews of the evidence indicate a failure on the part of governments and key decision-making bodies to account for long-standing gender inequalities in the design of health sector responses, as well as a failure of COVID-19 responses to address the norms, values, and power structures that underline persistent inequalities (5, 6).

Indian women, too, have suffered due to the pandemic with the loss of livelihoods, a steep increase in care work due to lockdowns, and a deeply patriarchal society that expects women to shoulder the brunt of unpaid domestic labor (7, 8). According to World Bank estimates, female labor force participation in India had already fallen from 30% in 1990 to 20.3% in 2019, and data released by the Ministry of Statistics and Programme Implementation (MoSPI) indicate that it fell further to 15.5% during April–June 2020 (9). As for the COVID effect, while in the initial months of the pandemic, more men lost jobs, male employment did recover with the unlocking of the economy, but the likelihood of being employed for women remained lower than that of men in mid-2020 (10). Women accounted for 73% of all job losses in April 2021, with a myriad of factors like digital divide, domestic responsibilities, lack of skill, and support contributing to the continued gender gaps in income and job losses (11). These findings are important as women’s participation rate in unpaid domestic service in India is about four times that of men, according to a 2019 national survey (10).

Of the many policy options for addressing gender inequities, gender budgets (GB) remain a key tool that countries have at their disposal. The historic patterns in gender inequalities all over the world have prompted the gender budgeting movement, which was seen as an important tool for empowering women and achieving gender equality. It was designed to address gender concerns along the complete policy cycle of planning, budgeting, implementation, and monitoring. While some countries, such as Australia and Canada, had already begun gender budgeting, the first push for GB at a global level came at the Fourth World Conference on Women in 1995 in Beijing, China. Most of the 189 countries that attended the meeting agreed to look at their national budgets through a gendered lens to integrate a gender perspective in their budgetary policies (12). The movement started with a somewhat narrow focus on improving the inequality between men and women, but has since expanded its scope and is now seen as a way of ensuring that budgets respond to the needs of all people. Over 80 countries have attempted to introduce methods and techniques of GB into their budgeting practices, to varying degrees of success (13). The most-used techniques are to include GB statements as sections within the primary budget documents (as in Uganda) or as an annexure to the main budget documents, published as a stand-alone report (such as India and Rwanda).

Given the further aggravation of gender inequalities during COVID, it stands to reason to ask if India has been able to respond to the aggravation of inequalities through gender budgets in the post-COVID period. In other words, whether and how budget allocations have changed to address gender concerns that the pandemic highlighted. To answer this question, we select two general health indicators for girls and women that have been key focus areas in government policies in India—Maternal Mortality rates (MMR) and anemia prevalence. Furthermore, we look at a more specific indicator of the wellbeing of women—the safety and security of women—in this study.

We begin the review by looking at some key indicators of gender gaps in India over the years, with a focus on the post-pandemic period in the next section. We also supplement this evidence in Section 3 with a brief literature review on violence against women in the post-pandemic period in India. In Section 4, we look at the latest trends in gender budgets of the central government and present our key findings. In the last section, we present our conclusions and recommendations.

## 2 Trends in key gender indicators in the post-pandemic period

We select three key indicators to examine the status and conditions of women in India: the global Gender Gap Index scores for India, the female labor force participation rate, and the health and wellbeing of women. Since GBs started much earlier than the start of the COVID pandemic, we look at trends over time with a focus on the post-pandemic period.

### 2.1 Global Gender Gap Index

The Global Gender Gap Index (GGI) was first published in 2006 by the World Economic Forum (WEF), and is designed to measure gender equality. It comprises four sub-indicators, which include Economic Participation and Opportunity, Educational Attainment, Health and Survival, and Political Empowerment. As of 2025, India is ranked 131st globally on the GGI out of 148 countries, and 5th within the South Asian region, with a score of 0.644, indicating that in India, the gender gap has only been partially met (14).

**Figure 1:**
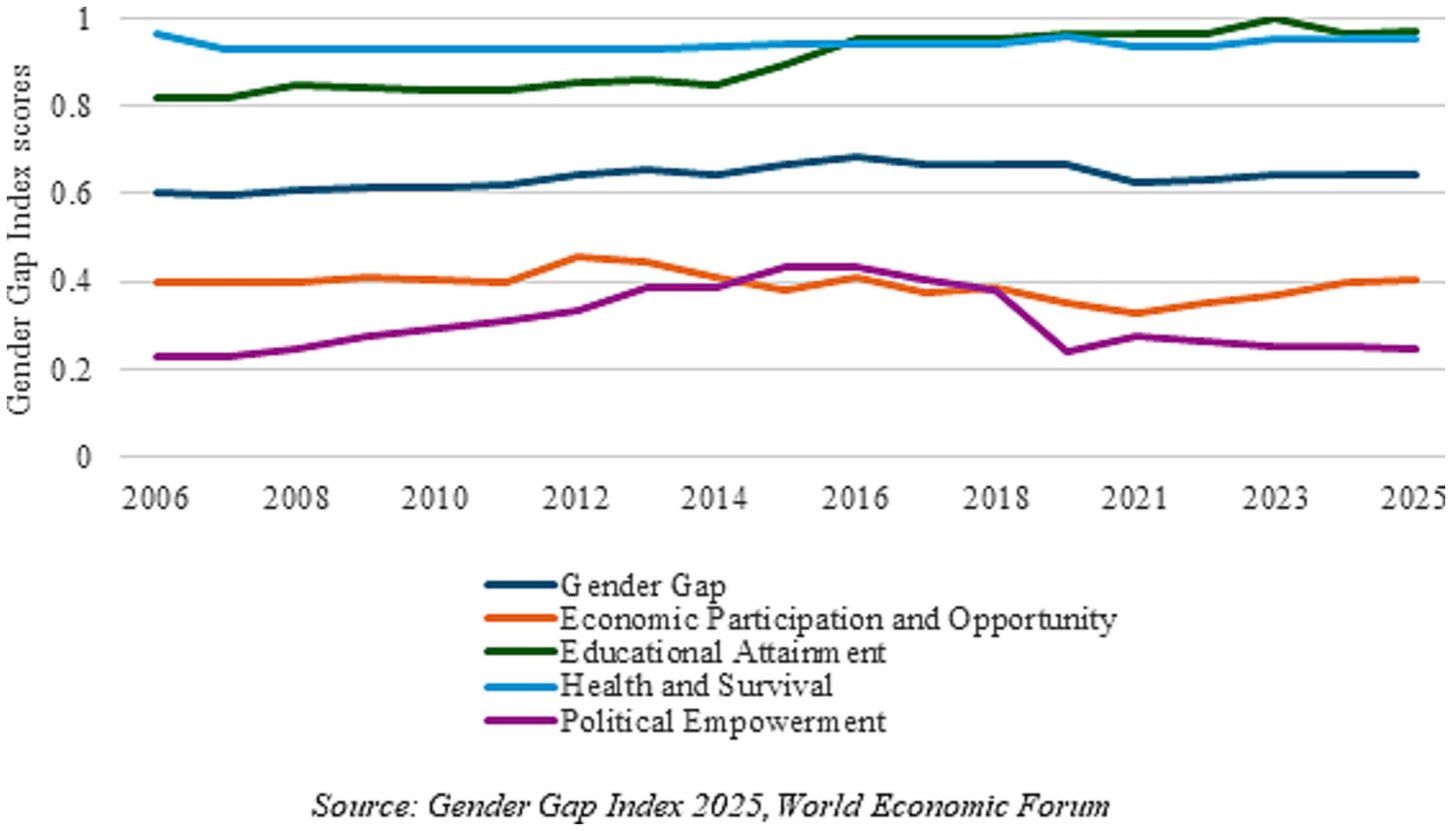
Indicators from the Gender Gap Index for India: 2006–2025. Source: Gender Gap Index 2025, World Economic Forum.

Figure 1 tracks India’s total scores on the GGI and its sub-indices over the period 2006–2025. The figure shows that there has been good progress made in the educational attainment gap—in fact, the improvement is the best in this indicator out of the four sub-indicators, and shows a rise even in the post-COVID period, though there has been a slight decrease in 2024 and 2025 as per the reports. There has been very little change in the health and survival domain; while the score shows that the gender gap has been 95.4% closed in this area, India ranks very low on this index, with only five other countries ranking lower, namely Bahrain, Israel, Vietnam, China, and Azerbaijan.

The two worst indicators are economic participation and opportunity, and political empowerment. The former has been falling over the last decade, with some slight improvements in the last 2 years, bringing it back to the level it was at in 2006. Moreover, while the political participation gap had narrowed initially, it has been going down since 2018 due to the reduced female representation in parliament and the lack of women ministers (14). These two indicators have greatly impacted the overall gender gap score. Overall, not much has changed over the years in the GGI—it was 0.6 in 2006 and is now only slightly more than that. While the COVID-19 pandemic in 2020 played some part in the recent value of the index, the stagnation has been since 2015–2016. India’s overall GGI score has dropped sharply in 2021 and has not managed to increase much since then.

### 2.2 Labor force participation rate

Another key indicator of women’s empowerment is the female labor force participation rate (LFPR). India is among the countries with the lowest LFPR for women (15). There have been debates and discussions around female LFPR in the recent period in India. It has been suggested that employment generation in the country has not kept up with the rise in the working-age population, due to increased competition with men for scarce jobs and an increasing reluctance of women to take up informal and lower-paid work. A related premise is that women have not benefited from the overall growth in employment opportunities due to industrial and occupational segregation (16, 17).

The female LFPR dropped by 10.1% from 2005 to 2010, indicating 22.6 million fewer women in the labor force, both in rural and urban areas (18). This has happened when India had been experiencing high GDP growth, a decline in fertility rates, an increase in higher education, as well as the initiation of the Mahatma Gandhi National Rural Employment Guarantee Scheme (MGNREGA) in 2005, so the lack of women in the labor force was surprising.

**Figure 2:**
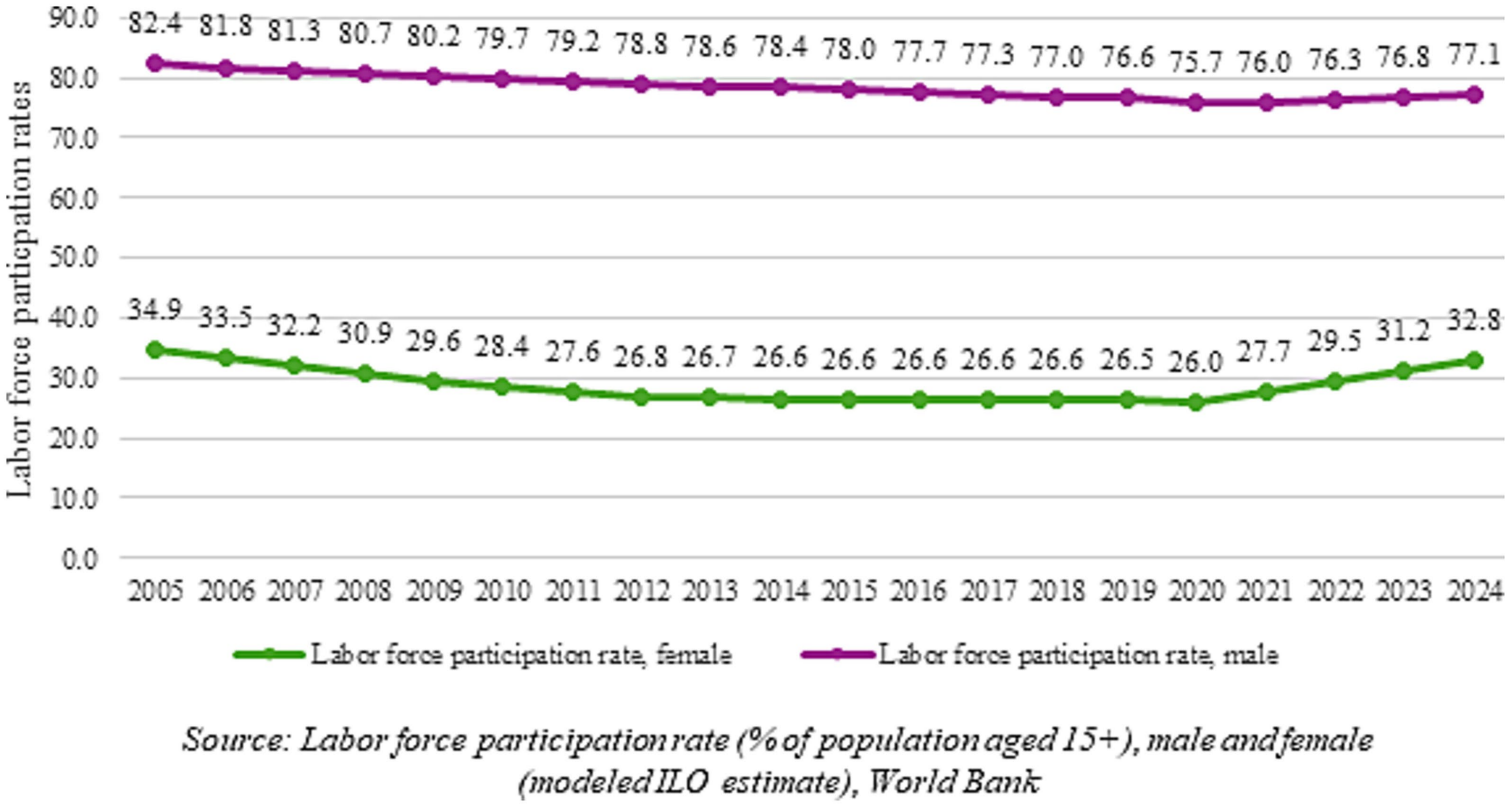
Male and female labor force participation rates in India: 2005–2024. Source: Labor force participation rate (% of population aged 15+), male and female (modeled ILO estimate), World Bank.

Data from the World Bank (Figure 2) indicate that the female LFPR had been decreasing slowly over the last decade, with the lowest rate of 26% during the first lockdown in 2020. However, it has been sharply increasing over the last few years, bringing it to 32.8% in 2024. The male LFPR has been declining as well and has not recovered to the 2005 level; despite this, the male LFPR remains much higher than the female LFPR. However, data from the Periodic Labour Force Survey Report 2023–2024 released by MoSPI shows that female LFPR has improved significantly to 42% in 2023–2024, as per the ‘usual status’ concept of measuring labor force participation. The government attributes this jump to its women-centric policy initiatives, including education, skill development, entrepreneurship facilitation, and safety in the workplace. However, it has been argued that this recent increase in female LFPR—especially in rural areas—between 2017–2018 and 2023–2024, based on government data, can be attributed to an increase in women in self-employment, which includes both paid work and disguised unemployment, especially in the absence of any increase in job availability in rural areas (10).

### 2.3 Health and wellbeing

A globally accepted indicator of women’s wellbeing is the Maternal Mortality Ratio or MMR, which is a key indicator in the Sustainable Development Goals (SDGs). Maternal deaths are almost entirely preventable, and according to the World Health Organization (WHO), Sub-Saharan Africa and Southern Asia accounted for approximately 87% of the estimated global maternal deaths in 2020. As per the report, the factors that prevent women from receiving or seeking care during pregnancy and childbirth are as follows: health system failures that include shortages of trained staff and medicines and poor quality of care, including disrespect, mistreatment and abuse; social determinants, including income, access to education, race and ethnicity, harmful gender norms and/or inequalities that result in a low prioritization of the rights of women and girls, including their right to safe, quality, and affordable sexual and reproductive health services; and external factors such as climate and humanitarian crises (19).

Thus, improvement in MMR is a key indication of positive changes in all these parameters that improve the status of women and their access to quality care.

Similarly, the general health of women is reflected in their anemia status, which often indicates general neglect of health and nutrition of adolescent girls and women. Anemia is one parameter that has historically been worse for girls and women globally and also for India, due to women’s physiological structure, including menstruation (20). In low- and middle-income countries, anemia is a major cause of morbidity among women of reproductive age (21). Studies have put forward several factors that contribute to anemia, including genetic conditions, prevalence of infectious diseases, nutritional deficiencies, and compromised environmental factors (22). There is evidence that suggests that lack of nutritious food and low access to healthcare services among the poor are major determinants of anemia in these countries (23). Unequal gender norms and social and cultural factors further worsen this situation in many countries, with women being expected to eat after everyone else in the family, often with an inadequate quantity and poorer quality food (24).

We will discuss these two indicators in the analysis later.

#### 2.3.1 Maternal mortality

**Figure 3:**
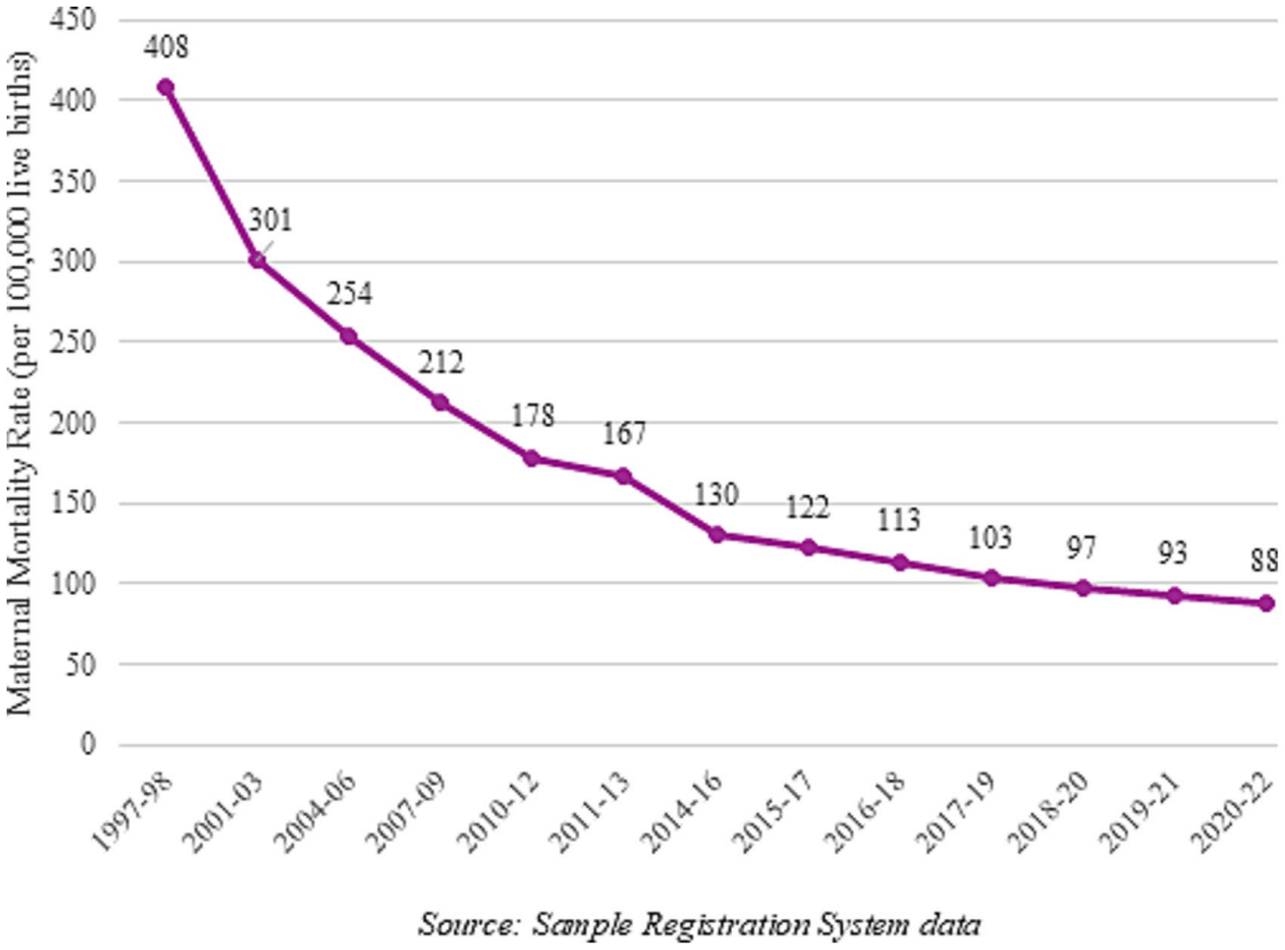
MMR (per 100,000 live births) in India from 1997 to 2022. Source: Sample Registration System data.

Figure 3 depicts the declining MMR in the country since 1997, as per numbers released by the Registrar General of India, based on the Sample Registration System (SRS) data.

MMR has been declining rapidly even before GB started; it used to be approximately 556 in 1990 and is now less than 100. Maternal health has been a key focus area of the government and the National Rural Health Mission, and subsequently, the National Health Mission (NHM) has had maternal health as one of the major themes for policy interventions. Schemes such as the *Janani Suraksha Yojana* and *Janani Shishu Suraksha Karyakram* under the NHM were also devised to help improve maternal and child health. The National Health Policy set a target of less than 100 deaths per 100,000 live births for the year 2020 and a target of less than 70 deaths per 100,000 live births for the year 2030, to coincide with SDG 3.1. Several states have already achieved this target for MMR, such as Kerala (MMR = 18) and Maharashtra (MMR = 36). However, there are at least 7 major states with MMR at much higher levels than the national average, such as Madhya Pradesh (MMR = 159), Chhattisgarh (MMR = 14), and Uttar Pradesh (MMR = 141) (25). Clearly, mere supply-side interventions have not proved sufficient to achieve a higher rate of decline in MMR. Gender norms and gender inequalities have acted as barriers preventing a higher rate of absorption of the various interventions.

MMR data is not available for the post-COVID period, so it is not possible to comment on post-COVID trends, but the fact that MMR started declining much before GBs started in India indicates that it was responding to other factors such as health investment, increase in education, and improvements in development indicators. The National Rural Health Mission (NRHM) was, in fact, focused on improvements in mother and child health, and did impact positively on maternal as well as child mortality (26).

#### 2.3.2 Prevalence of anemia

The prevalence of anemia among women and children in India has been a concern since the early 2000s (27). While 2008 WHO estimates suggested 24.8% of the global population suffers from anemia (28), in India, current estimates based on NFHS-5 state that 57% women and 67% children are affected by different types of nutritional anemia.

Table 1 shows the percentage of anemia prevalence in women and men, according to data from the National Family Health Surveys (NFHS). As can be observed, anemia prevalence is high among men too, but has been at a consistent 23–25% for many years, while it is much higher among women and has been increasing in recent years. The gap between men and women in anemia prevalence has remained more or less the same over the years.

**Table 1:** Percentage of anemia prevalence in women and men aged 15–49 years according to NFHS data (1998–2021).

Anemia prevalence	Women aged 15–49 years	Men aged 15–49 years
NFHS-5 (2019–2021)	57	25
NFHS-4 (2015–2016)	53.1	23
NFHS-3 (2005–2006)	55.3	24
NFHS-2 (1998–1999)	51.8	–

The government has initiated several national-level schemes working toward nutrition and anemia elimination over the years, including the National Nutritional Anemia Prophylaxis Program as early as 1970, the Integrated Child Development Services scheme in 1975, *Pradhan Mantri Matri Vandana Yojana* launched in 2010 and renamed in 2017, the Weekly Iron and Folic Acid Supplementation program launched in 2012, *Pradhan Mantri Surakshit Matritva Abhiyan* launched in 2016 and, and the *Anemia Mukt Bharat* program in 2018 (29). The *Saksham Anganwadi and Poshan 2.0* scheme, which started in 2021, also has significant budget allocations for anemia. However, this has not resulted in much change, as can be seen from Table 1, and on the contrary, anemia prevalence has been increasing in the last few years according to some researchers (30).

There are many suggested factors for the lack of progress on anemia. Iron folic acid (IFA) tablets are provided to all women under the *Anemia Mukt Bharat* program, but data show that distribution is far more than actual consumption (31), and that efforts involving distribution of food run into operational and logistical challenges, which make it difficult for the disbursements to reach the people who need them the most (32). Furthermore, lack of knowledge and information is a barrier as well, as the prevalence of anemia and the need for IFA tablets are frequently underestimated by women. Women are also socialized to take care of the nutritional needs of their families before taking care of their own, and in many places, girls/women are provided with less food than boys/men. Anemia prevalence is also found to be lower in women with higher education levels, or whose spouses have higher education levels, and in women belonging to higher wealth quintiles, indicating that access to better education and better resources is also an important factor for reducing anemia (33).

In sum, dealing with anemia required a more nuanced set of policies that looked at the social, cultural, and economic determinants of anemia prevalence among girls. Comparatively, addressing maternal mortality seemed somewhat easier, as it could respond to schemes like promoting institutional deliveries and regular antenatal check-ups, implemented by the government.

## 3 Violence against women and other gendered impacts of the COVID-19 pandemic in India

The WHO estimates that globally, 30% of women have been subjected to either physical and/or sexual violence (34).

Comparative evidence from the NFHS-4 and NFHS-5 finds that the percentage of young women (18–29 years) in India who have experienced sexual violence by the age of 18 years rose from 10.3% in 2014–2015 to 11.0% in 2019–2021, indicating an increase in domestic and sexual violence during COVID-19 pandemic-induced lockdowns (35).

A comprehensive analysis of crimes targeting women across various states and union territories in India from 2012 to 2021 was done using data from the National Crime Records Bureau (NCRB) (36). The offenses were segregated into three main categories: sexual crimes, domestic/dowry-related offenses, and other criminal acts. The results indicated that out of the 34 states, 12 states showed an increase in crimes between 2012 and 2021. States like Uttarakhand, Uttar Pradesh, Haryana, and Odisha saw large increases in crime rates against women.

Another analysis conducted across all districts of India using data from the NCRB on crimes against women for the period 2020 and 2022 indicated a rise in the reported crime against women nationally—the rate of crimes against women was 57 in the year 2020, and increased to 67 in 2022 (37). The national capital of Delhi had the highest crime rate at 145 in 2022.

Women and girls who experienced violence at home were trapped with the perpetrators during the COVID-19-induced lockdown, and there was a 131% increase in domestic violence complaints in May 2020 in districts that had stricter lockdown measures in comparison to districts that saw the least stringent lockdown measures (38). Furthermore, health systems were overwhelmed, leaving women with limited access to the prevention of GBV services. The increase in sexual violence also coincided with difficulty in accessing abortion services as well as contraceptive options (39).

Measures to mitigate the spread of COVID-19, such as quarantine orders, lockdowns, work-from-home arrangements, and school closures, especially threatened many women and girls for whom home was not a safe space (40). The already-existing gender digital divide led to women not having access to phones or computers, or not being able to use them safely at home for communication, which isolated them further, as they were dependent on male family members to access phone and internet-based services (41). Additionally, the harmful effects of the pandemic were compounded for women belonging to lower-caste groups as well as women living in rural areas, as they were found to have greater experiences of discrimination, higher exposure to intimate partner violence, more barriers in accessing healthcare services, and worse mental health outcomes (42).

**Figure 4:**
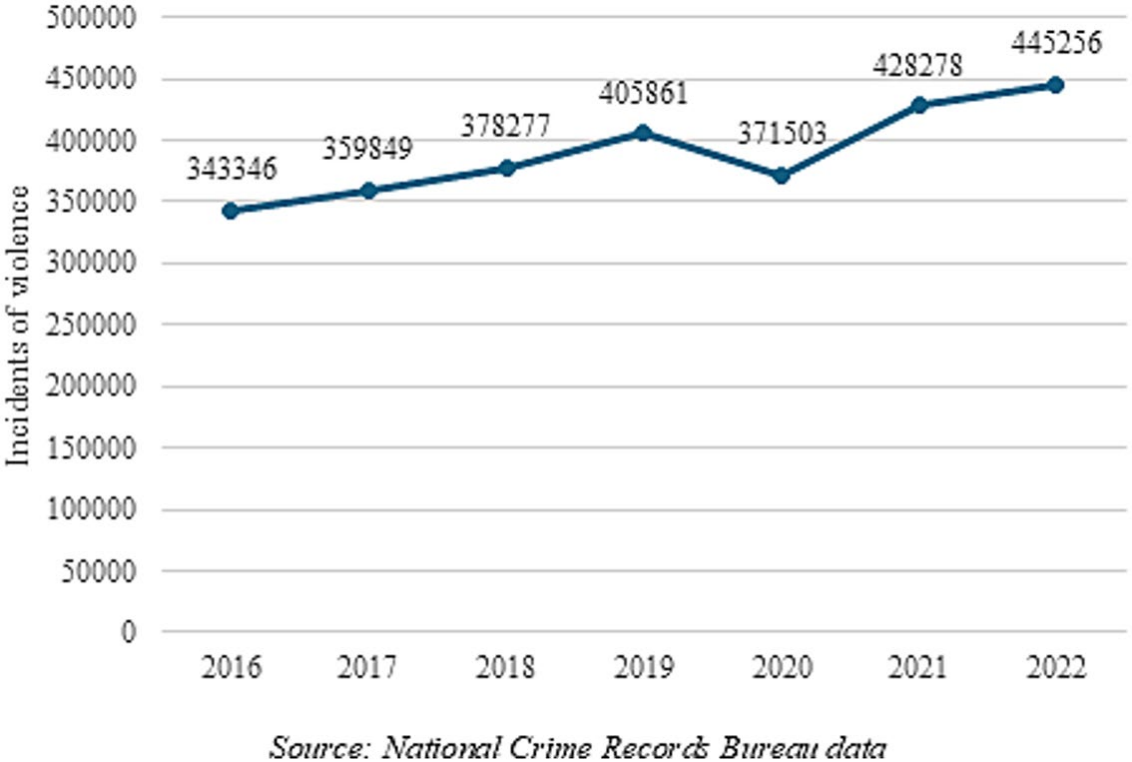
Incidents of violence against women in India as reported by the NCRB from 2016 to 2022. Source: National Crime Records Bureau data.

Figure 4 represents the total number of incidents of violence against women as reported by the NCRB from 2016 to 2022. Figure 4 shows a consistent rise in the reported incidents of violence against women during successive years, with the exception of 2020, which was the peak of COVID-related lockdowns. Overall, it is clear from the literature that women experienced greater risks of violence during the pandemic, and it is important to be vigilant even though the pandemic has abated (43).

## 4 Trends in gender budgeting in India

In the context of existing gender inequalities in India and COVID-related aggravations, an important question to ask is on budget allocations: did allocations for gender see an increase either during the pandemic or subsequent to the significant abating of the pandemic? This also raises an important question: do such gender-based allocations actually impact the parameters they are supposed to target?

One straightforward way of verifying this is to analyze the gender budget of a country. In this section, we analyze whether the GB of the central government in India since the COVID-19 pandemic adequately responded to the needs of women highlighted by the pandemic. In particular, we look at the trends in GBs over time to observe a departure from the trends, if any. The assumption is that any attempt at alleviating hardships and adverse impact on women cannot be brought about without a substantial increase in funding for the key women-specific components. Budget estimates show intent and are, therefore, important to understand policy focus. It must be noted that some states have their own GBs as well, but the central government’s role remains an important one, by leading in intent and allocations, and therefore, conveying its priorities to the states for possible adaptation at the state level.

India officially introduced gender budgeting in 2005 when the Expenditure Division of the Ministry of Finance (MoF) issued a note on Gender Budgeting as a part of the Budget Circular issued every year. But even prior to this, the Indian government had been incorporating elements of pro-women policies within the erstwhile Five-Year Plans (FYPs). For example, starting with the 5th FYP (1974–78), development and empowerment of women became an important focus, whereas earlier the focus had been on welfare for women (44).

In 2003, all ministries and departments were asked by the government to include a section on gender issues in their respective annual reports. The Ministry of Women and Child Development (MoWCD) was set up during this plan period. The following year, the MoF instructed all ministries to establish GB Cells, and from here on, ministries were expected to include a note in the budget circulars that would reflect gender allocations in two categories; Part-A: schemes that were targeted towards women with 100% of the budget allocation and Part-B: schemes where at least 30% of the budget was allocated for women (45).

Finally, in 2005–2006, the first Gender Budget Statement was released, which included budget statements from ten ministries, following the same Part-A and Part-B categorization. Each one was presented by the ministry in question and included a statement of the total provision of funds required for a given service, along with a statement of the detailed estimate of the grant divided into items. Since then, the GB statement has been released each year alongside the central government budget, with the same categorization of allocations (46). In addition to departments and ministries, some states have also incorporated GB into their budget planning. As of 2019, 57 Ministries and Departments had set up Gender Budget Cells to enable the integration of gender analysis into the budget, with an aim to end prevailing gender inequalities (47). In financial year 2024–2025, a third category was introduced—Part-C: schemes where less than 30% of the budget is allocated for women.

### 4.1 Overall trends

**Figure 5:**
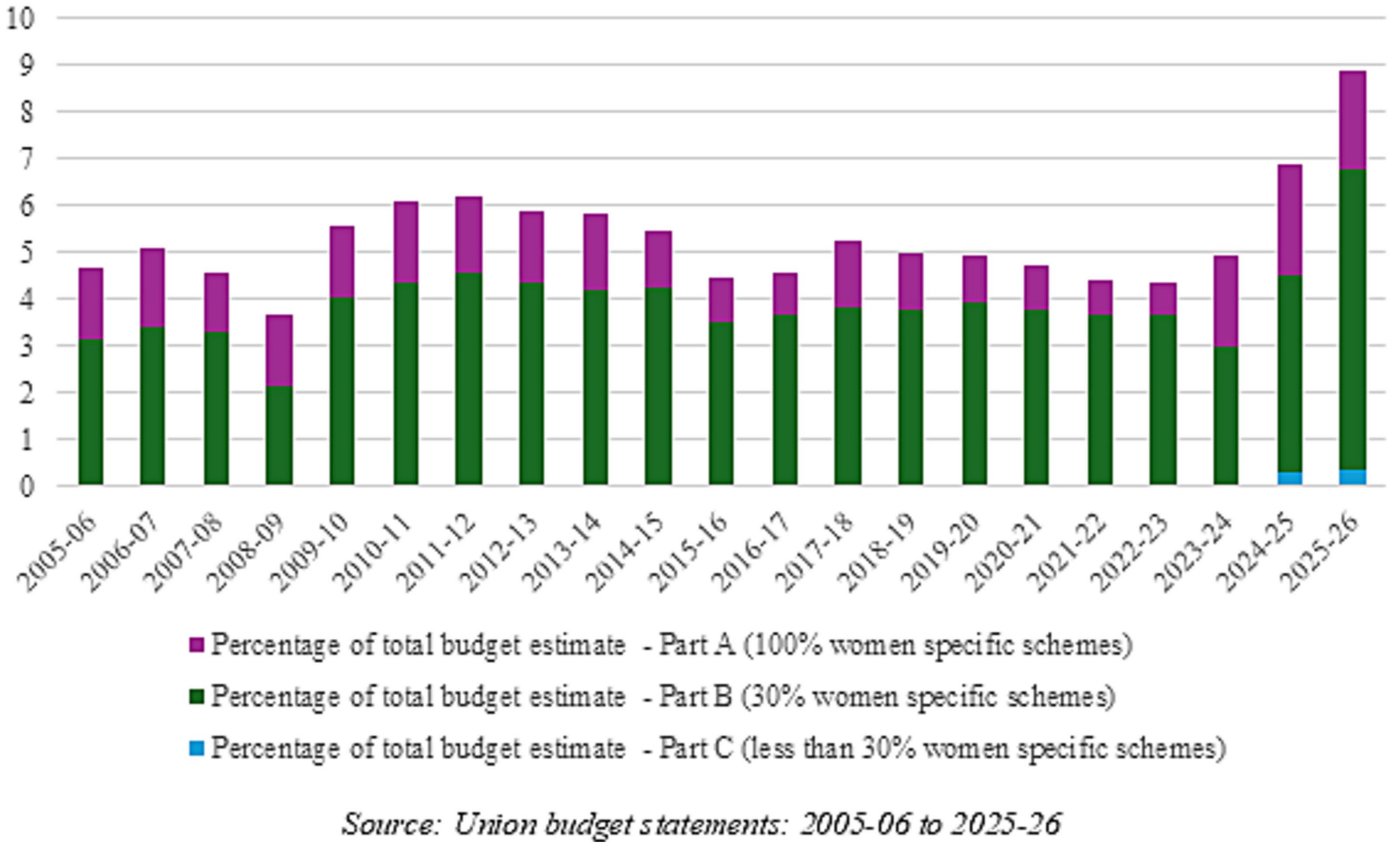
Gender budget estimates as a percentage of total budget for India: 2005–2025. Source: Union budget statements: 2005–2006 to 2025–2026.

Figure 5 shows the change in the allocations under budget estimates for Part-A, Part-B, and Part-C allocations over the last 20 years, between 2005 and 2025, as a percentage of the total budget allocation in that year.

Overall, GB allocations in total budget allocations had been increasing somewhat until 2012–2013, and since then, barring an increase in 2017–2018, it has seen an incremental decline until 2022–2023. The last 2 years have seen rising budget allocations with a sharp increase in the last two budgets for 2024–2025 and 2025–2026. The share of the gender budget has usually ranged between 4 and 6% of the entire central government budget estimate, but increased to 6.9% in 2024–2025 and then again to 8.9% in 2025–2026.

Furthermore, Figure 5 shows that the share of Part-A or 100% women-specific schemes has been declining over the years, and Part-B schemes have been increasing in the total—it is the major share of the GB. The 2023–2024 budget increased this share—it allocated 39.4% of the total gender budget in Part-A schemes and 60.6% in Part-B schemes, which was an increase of 229% from the previous year’s allocations for Part-A, and a decrease of 6% for the Part-B allocations. In the two subsequent GBs, the share of Part-A has largely remained constant, while Part-B allocations saw a steep increase in the 2024–2025 and 2025–2026 budgets. Additionally, the last two budgets also introduced budget allocations in Part-C, which currently comprise a very small percentage—0.33% of total budget allocations in 2025–2026. It remains to be seen what the revised and actual estimates would be; however, there seems to be a move toward fewer schemes and lower allocations entirely for women (Part-A), and more schemes with only partial allocations for women (mainly Part-B).

**Figure 6:**
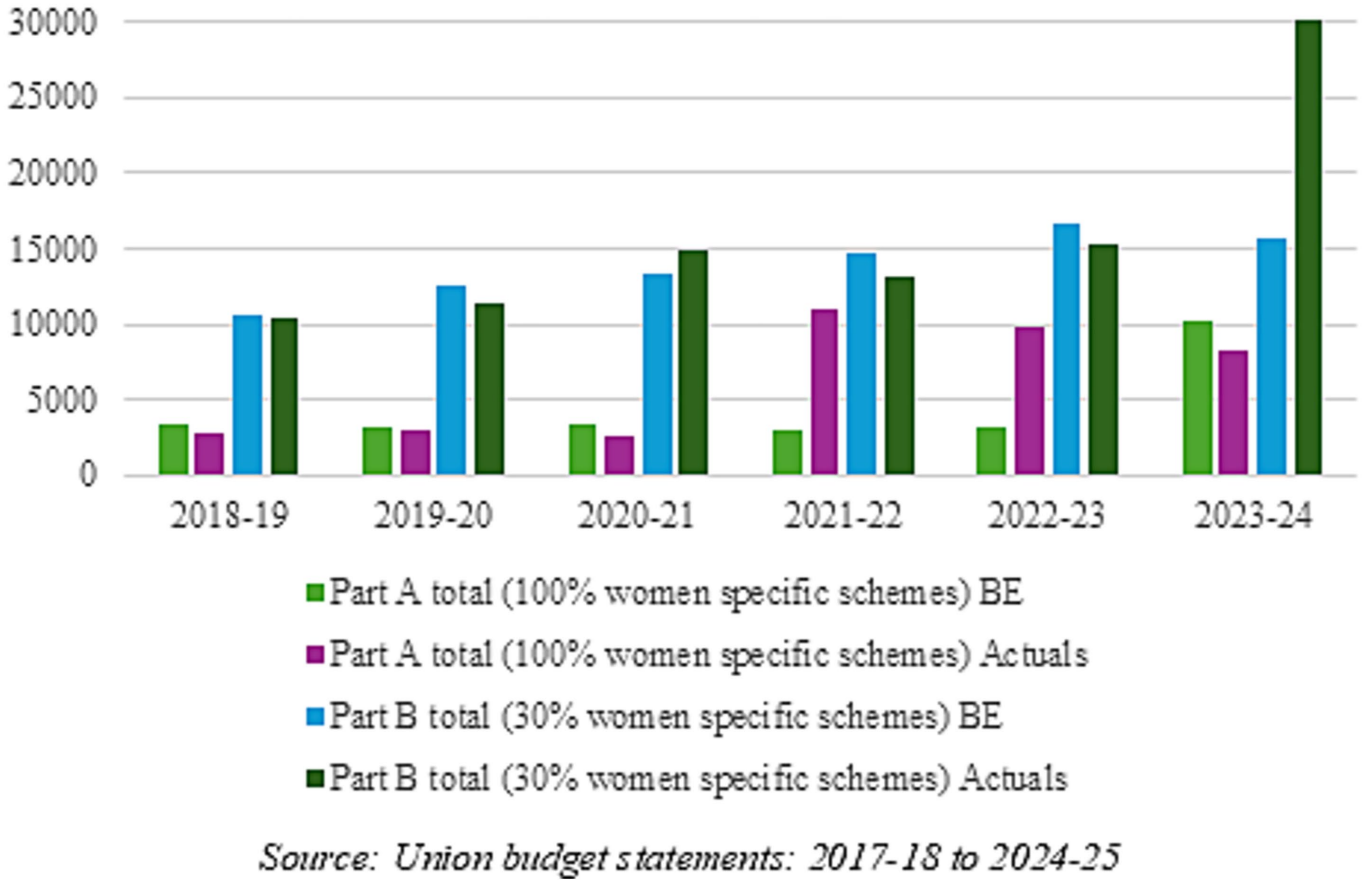
Gender budget estimates vs. actuals for 2017–2018 to 2022–2023 (in million USD). Source: Union budget statements: 2017–2018 to 2024–2025.

Figure 6 depicts the budget allocations and actual expenditure for the years 2018–2019 to 2023–2024 for Part-A and Part-B to indicate that actual expenditures often deviate significantly from budgeted and revised estimates. While actuals have been slightly lower than budget estimates during 2017–2020, actuals for Part-A sharply increased in 2021–2022 and in 2022–2023, which is likely partially responsible for the increase in budget allocations for 2023–2024 and 2024–2025. Similarly, Part-B actuals increased sharply in 2023–2024, leading to the large increase in Part-B allocations in subsequent budgets.

In terms of its overall importance, the GB has usually been slightly less than 1% of the total GDP.

### 4.2 Major features of the post-pandemic gender budgets

Table 2 depicts the ministries and departments with the largest budget allocations (all three parts included), based on the GB statements from 2020–2021 to 2025–2026.

**Table 2:** Percentage of budget allocations for key ministries and departments from 2020–2021 to 2025–2026.

Ministry/Department	2020–2021	2021–2022	2022–2023	2023–2024	2024–2025	2025–2026
Department of Rural Development	32%	34%	32%	40%	33%	27%
Department of Food and Public Distribution					5%	24%
Department of Health and Family Welfare	19%	20%	18%	14%	11%	9%
Department of School Education and Literacy	12%	10%	10%	10%	8%	6%
Ministry of Housing and Urban Affairs	2%	5%	14%	11%	8%	5%
Ministry of Women and Child Development	14%	11%	9%	9%	6%	5%
Department of Drinking Water and Sanitation		2%	1%		11%	5%
Department of Higher Education	8%	8%	8%	7%	5%	4%
Department of Agriculture and Farmers Welfare	3%	3%	1%			4%
Others	10%	7%	7%	10%	12%	10%

As can be seen from Table 2, major allocations for GB have been for only a few ministries and departments. Ministries of Rural Development, Health and Family Welfare, School Education and Literacy, Housing and Urban Affairs, and Women and Child Development have significant allocations within the GB. In the most recent 2024–2025 and 2025–2025 GBs, some additional ministries/departments showed large allocations, including the Ministry of Drinking Water and Sanitation and the Department of Food and Public Distribution. It is safe to say, therefore, that the focus of GB is on rural development, health and nutrition, housing, education, and livelihoods. However, it can also be observed that the percentage of GB allocations for health, education, and even the MoWCD have been decreasing, while those for the provision of housing and public services have been increasing.

Housing and livelihood schemes comprise a very large chunk of the GB. In the GB of 2023–2024, the two *Pradhan Mantri Awaas Yojana* (PMAY) schemes*—*rural and urban—under the Ministry of Rural Development (MoRD) and the Ministry of Housing and Urban Affairs (MoHUA), respectively, together comprised 36% of the overall gender budget. The PMAY is a housing scheme that provides interest subsidies to beneficiaries applying for home loans or financial assistance for the construction of houses in rural areas. The allocations for these schemes have remained high in the 2024–25 and 2025–2026 GBs, though the percentage has decreased due to additional ministries reporting allocations.

As the discussion in the previous section indicated, women’s employment plays a key role in women’s empowerment. Several schemes focused on employment, livelihoods, and skilling of women reported major allocations in the GBs, including MGNREGA and National Rural Livelihood Mission—*Aajeevika* under the MoRD, *Deendayal Antyodaya Yojana*—National Urban Livelihoods Mission under MoHUA, as well as several programs under the MoWCD.

Health and nutrition together have seen major allocations including *Saksham Anganwadi and Poshan 2.0* under MoWCD, which attempts to tackle the challenges of malnutrition in children, adolescent girls, pregnant women and lactating mothers, the *Pradhan Mantri Poshan Shakti Nirman* under the Department of School Education and Literacy which provides mid-day meals to school children, and the *Pradhan Mantri Garib Kalyan Anna Yojana* under Department of Food and Public Distribution aimed at providing food grains to poor people. All of these programs try to reduce the burden of anemia and malnutrition, and allocations for the programs have increased every year. Similarly, the flexible pool for reproductive and child health has seen sharply increasing allocations each successive year under NHM.

**Figure 7:**
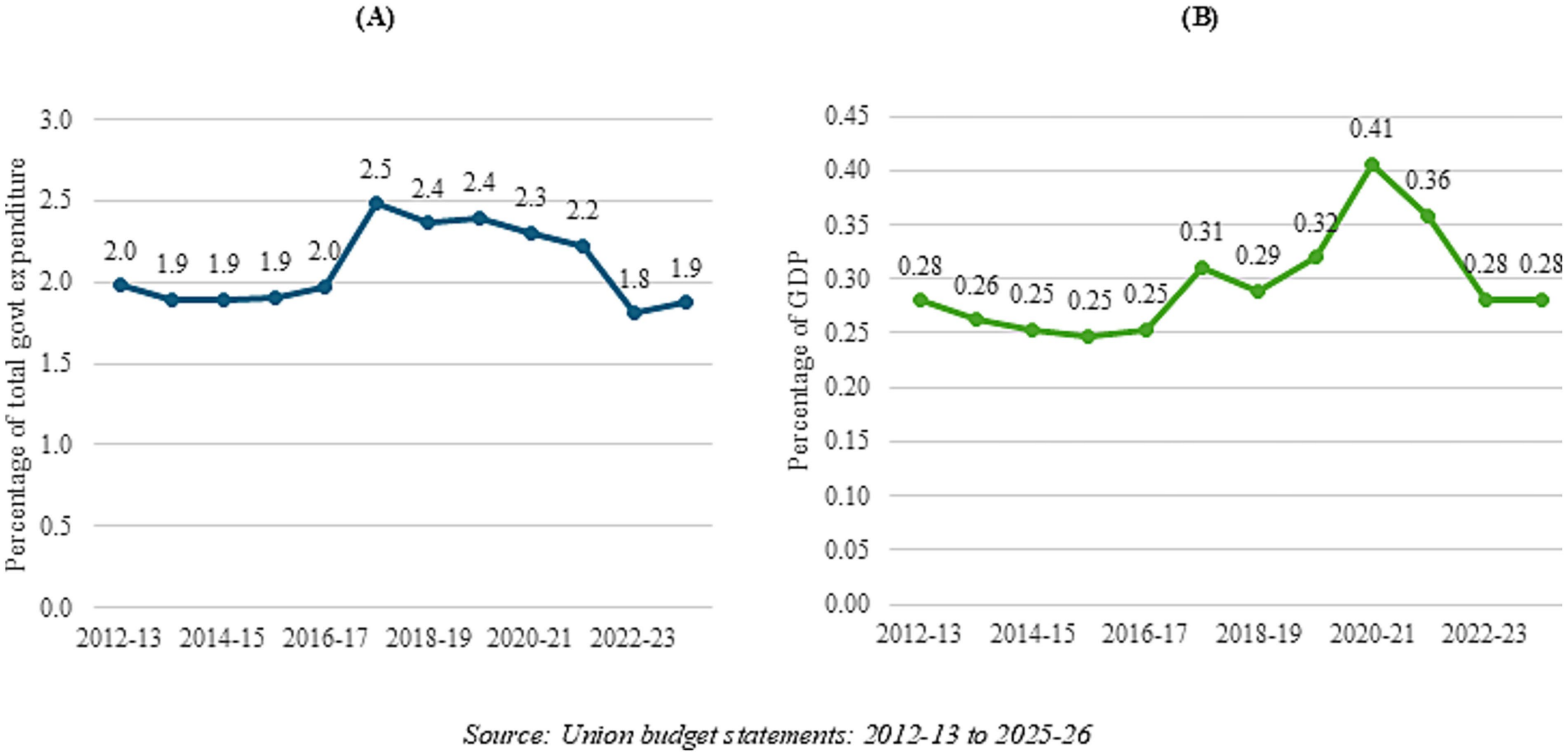
Government health expenditure from 2012-2013 to 2023-2024 as a percentage of **(A)** total government expenditure, and **(B)** GDP.

However, these trends need to be seen in the context of the overall spending of key ministries and departments. There has been no perceptible increase in the health budget out of either the total budget of the government or out of GDP in India in the post-pandemic years. Figure 7A looks at actual spending of the Ministry of Health and Family Welfare (MoHFW) as a percentage of total government expenditure, and Figure 7B tracks actual spending of the MoHFW as a percentage of India’s GDP. As can be seen, there has not been any increase in the share of the health sector in the budget either out of total expenditure or out of GDP; in fact, there has been a fall in the share in total expenditure since 2017–2018. Health expenditure of the central government has stayed at less than 2% of total government expenditure even after the pandemic, and remains at approximately 0.31% of India’s GDP.

For addressing maternal and child deaths, the MoHFW has been running a number of programs and interventions over the years. The National Health Mission (NHM) is a flagship program of the ministry, which has been running since 2005 on a center-state sharing basis, co-terminus with the launch of GB. The focus of NHM has been on mother and child health and has been seen as a successful program despite some challenges (48). NHM is also globally known for its conditional-cash transfer program, the *Janani Suraksha Yojana*. It stands to reason that a large part of the NHM allocations are, in fact, able to address reproductive and child health. However, the GB does not reflect this in Part-A, and has some allocations in Part-B under different heads. In the face of unchanging government allocations for the health sector post-COVID, and in fact some reduction in the allocation to the NHM in recent budgets, it stands to reason that the GB for the health sector remains nominal, even if NHM is counted in Part-A of the GB. Thus, even though the GB allocations look higher, the actual allocations would remain modest enough not to make too much of a difference.

Similarly, a study that takes a closer look at the GB of MoWCD—a key ministry for addressing women’s concerns—found that though the budget allocations have been increasing and the ministry’s expenditure has grown annually at a rate of approximately 4% between 2015–2016 and 2025–2026, MoWCD’s expenditure as a share of total central government expenditure declined from about 1% in 2015–2016 to 0.5% in 2025–2026 (49).

There are several key areas where allocations for women are much needed, but are not being given enough focus, such as digital literacy and violence against women and girls. The Safe City Projects program under the Police department introduced in 2022–2023, which aims to create a safe, secure and empowering environment for women in public places and eliminate the threat of gender-based violence and/or harassment, saw a large increase from USD 60 million to USD 150 million in 2023–2024, but was cut down to USD 24.8 million in 2024–2025 and 2025–2026. The *Nirbhaya Fund* under the Police Department was brought back in the 2024–2025 GB, but the allocation was only USD 5.8 million, which was raised to USD 23.1 million in 2025–2026.

The Mission Shakti program, introduced in 2021–2022 under the MoWCD, has two verticals—*Sambal,* focusing on safety and security for women, and *Samarthya,* focusing on empowerment of women. The *Sambal* scheme combines the One Stop Centres, Women Helplines, *Nari Adalats,* and *Beti Bachao Beti Padhao* programs, and allocations for this scheme increased from USD 64.9 million in 2023–2024 to USD 72.7 million in 2024–2025, and remained the same in 2025–2026. The *Samarthya* scheme comprises livelihood programs and *Shakti Sadan,* which offers rehabilitation to women in difficult circumstances. While the allocations for *Samarthya* are much higher, at USD 294.2 million in 2022–2023, they have been steadily decreasing in subsequent GBs, by 2% in 2023–2024, then an additional 5% in 2024–2025, and then increased by 1% to USD 276.7 million in 2025–2026.

Women also lag far behind men when it comes to digital skills, and lose out on online education, health, and employment opportunities (50). In terms of the gender budget, however, only a small allocation of USD 13.9 million was made for rural digital literacy under the Ministry of Electronics and Information Technology (MEITy) in the *PM Gramin Digital Saksharta Abhiyan* scheme in 2021–2022, which declined by 17% to USD 11.6 million in 2022–2023, and allocations were discontinued in 2023–2024. In its place, the allocation of USD 11.6 million was given to the Capacity Building and Skill Development Scheme under the MEITy, and increased to USD 31.1 million in 2024–2025. The Digital India program under MEITy was also discontinued in 2022–2023. In 2023–2024, the Skill India program, with an allocation of USD 263.1 million, was introduced in the gender budget under the Ministry of Skill Development and Entrepreneurship, though building digital skills is not the only focus of this program. Its stated objectives are to provide a unified platform enabling demand-based formal skilling, linking with employers, including Micro, Small, and Medium Enterprises (MSMEs), and facilitating access to entrepreneurship schemes, which are important objectives but do not focus on helping women gain digital skills. However, in the 2024–2025 budget, allocations for this program were slashed to USD 88.2 million as well, and then further to USD 72.3 million in 2025–2026.

Overall, there have been some good initiatives under GB allocations, but since the total allocations have remained more or less static, it is doubtful if these would be sufficient to alleviate the concerns of health, safety, and security of women that were brought into focus by the pandemic.

### 4.3 Limitations of the gender budget

Numerous experts have analyzed India’s GB efforts and given credit to India for the early start made in institutionalizing the GB statement, setting up GB cells, and the engagement and overall direction given by the MoF to streamline the process (51). However, there remain several limitations to the GB statement, like methodological inaccuracies, errors in the classification of allocations, missing allocations, double counting of grants, difficulty in accounting for separation in allocations by gender, and lack of sex-disaggregated data for analysis and measurement of progress (52). There are several women-focused schemes that are not mentioned in the gender budget, including the newly announced schemes like the *Lakhpati Didi* scheme or the *Mahila Samman* Saving Certificate program. Many ministries and departments, including the Ministry of Finance, the Department of Telecommunications, and the Ministry of Law and Justice, among others, do not report any allocations under the gender budget. Even the MoHFW does not accurately report on allocations that are meant mostly for women.

In the last few budgets, schemes have been moved from Part-A to Part-B or vice versa, though the beneficiaries of these schemes have not been changed, indicating that not enough attention is given to whether a scheme is completely or partially targeted to women. For example, PMAY (both rural and urban) is a housing subsidy scheme under which subsidies on home loan rates are provided to borrowers, along with special interest rates and benefits offered to women. Furthermore, ownership of the home is meant to be under the woman’s name alone, or jointly under the man’s and woman’s names, or in cases where there is no woman in the family, under a man’s name. According to data shared by the government in August 2023, 69% of the houses constructed under PMAY (rural) are owned either wholly or jointly by women (26% by women outright and 43% jointly along with husbands) (53). Thus, it is unclear why this scheme is mentioned under Part-A allocations, as a significant number of beneficiaries are men as well. Similarly, the *Aajeevika* program is a poverty alleviation program implemented by MoRD in 2011 with women as one of the key beneficiary groups, but not the only one, and it was also moved to Part-A allocations last year, despite not being 100% targeted to women.

Another limitation mentioned is the issue of fiscal marksmanship (54). Fiscal marksmanship is the accuracy of budgetary forecasting, or the difference between the budget estimates and the actual spending. The study finds a large number of schemes overestimating their budget allocations, often leading to underutilization of funds. The converse is also true, with several schemes underestimating budgetary allocations, leading to a lack of funding in required areas.

Given these various limitations, the GB statement remains a statement of *ex-post* allocations. Most importantly, there is no analysis of the source or cause of gender inequality, nor the impact of the various schemes and programs on relevant indicators.

These trends and challenges have persisted during the COVID years as well as the post-COVID period. There have not been any changes in the approach to GB, keeping in view the challenges faced by women during COVID. The allocations on the safety and security of women have been surprisingly modest and, in fact, reduced in the last few years. Similarly, health ministry allocations have remained almost static out of India’s GDP, and schemes meant for women—whether for reproductive health or for reducing anemia—have not been significant enough to bring about a faster improvement in the health of women.

Finally, it does seem as though the GB exercise has been relegated to a routine administrative one, with neither vision nor clear objectives; it remains another box that needs to be ticked for budget purposes.

## 5 Discussion and recommendations

The analysis throws up some important questions. Over the years, progress has been made in education and some key health areas for women, but the improvements have been uneven and at times modest. LFPR and political empowerment of women have, in fact, declined.

MMR, as also infant and child mortality (not looked at in this study), have responded to investments and interventions under the MoHFW via programs like the NRHM/NHM—irrespective of their subsequent categorization as gender investments. It is probably safe to say that a higher investment in health would have brought about an even faster rate of improvement—evidence abounds on the returns to investment in health. Government (center plus states) spending on health currently stands at a meagre 1.8% of its GDP (55); it stands to reason that with higher health allocations, outcomes would have improved for both genders. India is still grappling with various women-specific issues like maternal deaths and anemia among girls and women, but has been able to address these to some extent via many of the government programs running in the country. This indicates that areas that are amenable to health interventions via health budget allocations, including health system strengthening, are easier to impact, but outcomes that are linked more to awareness, attitudes, and social mores, including discrimination, are harder to impact through mere routine budget allocations. This is also true of labor market participation and political empowerment. These areas require additional budgets, but only if based on needs assessment and evidence-based allocations. In fact, GBs would be extremely useful in implementing innovative and novel schemes for women, which are currently not part of routine allocations of key ministries like MoHFW and MoWCD.

For example, a 2022 study based on NFHS-4 on decision-making, economic empowerment, and other socioeconomic variables of currently married women found that economic empowerment was significant in improving women’s decision-making abilities and made it easier for women to negotiate complex family and societal norms and values (56). Another study based on NFHS data confirmed that economically empowered women were less likely to face violence from mothers, fathers, and other family members and that in healthcare decisions, women’s empowerment correlated with reduced violence (57). However, economic empowerment continues to evade most Indian women. While MGNREGA has been a significant source of employment and earnings for many women, an analysis and review indicate that MGNREGA has not really addressed the constraints faced by women when they participate in the schemes (58). Specifically, the implementation of MGNREGA is predicated on the current political and social gender norms that prevent women from lending a voice to their concerns, like a lack of support for childcare and household responsibilities. The review also indicates that though women make up a sizeable part of total employment under this scheme, it is probably due to a lack of alternatives and also a shift from other kinds of unpaid or informal sector work to MGNREGA.

To actually make a difference to women’s empowerment, one has to go beyond mere participation statistics and look at the needs, concerns, and challenges faced by women in participating in the labor market. Employment generation for women with emphasis on newer modern occupational choices remains a weak area in India’s policymaking. Looking at the allocations for employment, other initiatives mentioned in the GB are National Rural Livelihood Mission—*Aajeevika* under MoRD, National Child Labor Project, Labor Welfare Scheme, and *Pradhan Mantri Shram Yogi Maandhan* under the Ministry of Labor, and *Deendayal Antyodaya Yojana*—National Urban Livelihoods Mission under MoHUA. However, all of these programs except *Aajeevika* come under Part-B of the GB, indicating that their focus is not solely on women. Even *Aajeevika*—which has now been included under Part-A allocations—is not solely targeted at women, and in any case, allocations remain low for all of them. Basically, women continue to have little scope for gainful employment in India, which is also corroborated by other studies (10). The GB, as an additional tool with the government, could have addressed this by creating newer and more modern job opportunities for women, which it has not been able to do.

Another key consideration is that a lot of the central government’s budget allocations for important ministries like the MoHFW and MoWCD are meant for centrally sponsored schemes, which are implemented by state governments. The results and conclusions around the central government’s GB also hold for the state governments, as evidenced by the fact that often the states are unable to utilize the funds allocated to them and show significant unspent balances, leading to further lowering of budget allocations to key schemes of the center (49).

As for political participation, in India, the effort has been to increase women’s political participation mainly through mandates. However, it has been argued that the number of women contesting parliamentary and state legislative elections remains limited, mainly due to political parties being inaccessible to women (59). That the battle for women’s political participation continues to be grim can be seen from the fact that the Women’s Reservation Bill 2008, which proposes mandated reservation of one-third of parliamentary and state assembly seats for women, continues to face stiff resistance.

This again brings out a key point about improving women’s wellbeing in India. While mandates and regulations can work to a certain extent, progress will remain unsatisfactory unless transformation happens in societal norms and prejudices. The shortcomings of implementation and monitoring of MGNREGA, in fact, bring out the shortcomings of the GB approach. In its current format, the GB has little scope for evidence-based informed policymaking and ends up being another routine administrative requirement that ministries and departments have to meet. Interventions have to be evidence-based and novel, and strike areas that have been resistant to change. Much of this would be behavioral interventions requiring different types of ‘nudges’ and will require budget-based funding. The current allocations are neither sufficient nor novel for bringing about those changes. There is substantial scope in GBs to bring about such transformations if policymakers truly want to make a difference to women’s health, education, social, economic, and political status. For that, out-of-the-box thinking would be required and a willingness to work with communities and grassroots organizations that work with women; these bodies are in touch with the realities of women’s lived experiences and can help bring about the required changes by focusing on areas that are likely to be missed by top-down interventions.

Till that happens, the GB exercise would remain a blunt and somewhat irrelevant tool for improving the status of women in India.
